# Declining HIV incidence in sub‐Saharan Africa: a systematic review and meta‐analysis of empiric data

**DOI:** 10.1002/jia2.25818

**Published:** 2021-10-20

**Authors:** Keya Joshi, Justin Lessler, Oluwasolape Olawore, Gideon Loevinsohn, Sophrena Bushey, Aaron A R Tobian, M Kate Grabowski

**Affiliations:** ^1^ Department of Epidemiology Harvard TH Chan School of Public Health Boston Massachusetts USA; ^2^ Department of Epidemiology Johns Hopkins Bloomberg School of Public Health Baltimore Maryland USA; ^3^ Johns Hopkins University School of Medicine Baltimore Maryland USA; ^4^ Department of Pathology Johns Hopkins University School of Medicine Baltimore Maryland USA

**Keywords:** Africa, clinical trials, cohort studies, HIV epidemiology, HIV incidence, HIV prevention, key and vulnerable populations

## Abstract

**Introduction:**

UNAIDS models suggest HIV incidence is declining in sub‐Saharan Africa. The objective of this study was to assess whether modelled trends are supported by empirical evidence.

**Methods:**

We conducted a systematic review and meta‐analysis of adult HIV incidence data from sub‐Saharan Africa by searching Embase, Scopus, PubMed and OVID databases and technical reports published between 1 January 2010 and 23 July 2019. We included prospective and cross‐sectional studies that directly measured incidence from blood samples. Incidence data were abstracted according to population risk group, geographic location, sex, intervention arm and calendar period. Weighted regression models were used to assess incidence trends across general population studies by sex. We also identified studies reporting greater than or equal to three incidence measurements since 2010 and assessed trends within them.

**Results:**

Total 291 studies, including 22 sub‐Saharan African countries, met inclusion criteria. Most studies were conducted in South Africa (*n* = 102), Uganda (*n* = 46) and Kenya (*n* = 41); there were 26 countries with no published incidence data, most in western and central Africa. Data were most commonly derived from prospective observational studies (*n* = 163; 56%) and from geographically defined populations with limited demographic or risk‐based enrolment criteria other than age (i.e., general population studies; *n* = 151; 52%). Across general population studies, average annual incidence declines since 2010 were 0.12/100 person‐years (95% CI: 0.06–0.18; *p* = 0.001) among men and 0.10/100 person‐years (95% CI: −0.02–0.22; *p* = 0.093) among women in eastern Africa, and 0.25/100 person‐years (95% CI: 0.17–034; *p* < 0.0001) among men and 0.42/100 person‐years (95% CI: 0.23–0.62; *p* = 0.0002) among women in southern Africa. In nine of 10 studies with multiple measurements, incidence declined over time, including in two studies of key populations. Across all population risk groups, the highest HIV incidence estimates were observed among men who have sex with men, with rates ranging from 1.0 to 15.4/100 person‐years. Within general population studies, incidence was typically higher in women than men with a median female‐to‐male incidence rate ratio of 1.47 (IQR: 1.11 to 1.83) with evidence of a growing sex disparity over time.

**Conclusions:**

Empirical incidence data show the rate of new HIV infections is declining in eastern and southern Africa. However, recent incidence data are non‐existent or very limited for many countries and key populations.

## 1 INTRODUCTION

Nearly 40 years into the HIV pandemic, sub‐Saharan Africa remains the global epicentre of HIV transmission, accounting for 57% of all new infections in 2019 [1]. Within most African countries, transmission continues to be generalized with substantial rates of new infections outside of clearly defined risk groups, particularly in eastern and southern Africa [1]. Widespread community transmission within the region has spurred substantial investment in population‐based infection control measures, including biomedical and behavioural interventions. Domestic and international donors, including the Global Fund to Fight AIDS, Tuberculosis, and Malaria and the United States President's Emergency Plan for AIDS Relief (PEPFAR), continue to support HIV programming in sub‐Saharan Africa with significant financial and human resources investment in large‐scale HIV testing, prevention and treatment programmes. However, global funding for HIV programming within Africa is waning, potentially jeopardizing progress towards UNAIDS fast‐track goals to end the African epidemic by 2030 [2]. Measuring the impact of programmatic investments to control HIV is critical for demonstrating headway, appropriately targeting future funding and other resources, and ensuring a sustained global commitment to ending the HIV pandemic.

Key epidemiologic metrics of HIV control include absolute rates of HIV incidence and AIDS‐related deaths and percentage reductions in new infections and AIDS‐related deaths among other benchmarks [3, 4]. While each of these control criteria provides key insights into epidemic trajectories, HIV incidence is arguably the most important of these metrics because it is less impacted by fluctuations in non‐HIV mortality and more comparable across different populations and geographic settings [4]. However, directly observed HIV incidence measurements are difficult to obtain, partly due to delays in testing following infection, limited availability of testing to detect early HIV infections and difficulty enumerating and following populations at risk. Thus, data on HIV incidence trends in sub‐Saharan Africa predominantly come from UNAIDS mathematical models, which use demographic, HIV prevalence and programmatic data [5, 6, 7, 8]. Based on these models, UNAIDS reports that HIV incidence is steadily declining across sub‐Saharan Africa. However, there are limited direct empirical data supporting these modelled downward trends. The last comprehensive systematic review of the empiric HIV incidence literature from sub‐Saharan Africa was published in 2009 [9]. This prior review, including only 57 studies, found that HIV incidence data from western and central Africa were rare and that estimates varied substantially within countries and across population risk groups: no trend data were analysed.

Here, we provide an update on empirical HIV incidence data published through 2019. Our systematic review and meta‐analysis includes all studies reporting directly observed HIV incidence measurements obtained using serologic assays either through prospective or cross‐sectional assessments. Data were stratified by gender and risk group where possible. Regional variations in incidence data are reported along with trends over time across and within studies, by sex and across various population risk groups, including men who have sex with men (MSM) and sex workers.

## 2 METHODS

### 2.1 Screening and extraction protocol

We performed a systematic literature review and meta‐analysis to identify studies on HIV incidence from sub‐Saharan Africa published between 1 January 2010 and 23 July 2019. The review was completed in July 2020 and the analysis in October 2020. We searched PubMed, Embase, Scopus and OVID global health databases for peer‐reviewed articles reporting directly observed (i.e., empirical) estimates of HIV incidence measured through either prospective repeat testing or cross‐sectional HIV incidence testing of blood samples. We used the following search terms “HIV”, “incidence” and “Africa” as medical subject heading (MESH) terms. Additional clinical synonyms and alternative spellings were also included in the search (see Supporting Information files for full search criteria). Technical reports measuring population‐level HIV incidence, but not published in peer‐reviewed journals, such as the population‐based HIV impact assessment (PHIA) surveys, were also included.

Our analysis included peer‐reviewed studies and technical reports including national or subnational empiric estimates of HIV incidence from sub‐Saharan Africa. We excluded studies of HIV not in humans, studies reporting incidence estimates for children <15 years of age only, studies not in English, perspectives, opinions, commentaries, non‐nested case control studies, studies measuring impact of postexposure prophylaxis among healthcare workers (HCW), cross‐sectional incidence studies only including HIV‐positive persons and incidence estimates based on mathematical models. We further excluded studies with incidence estimates based on less than 50 total participants or less than 50 person‐years (pys) at risk.

After removal of duplicates, all studies identified through the database search were uploaded to the Covidence systematic review management software and then screened by two independent reviewers (K.J., S.B., G.L., O.O.) [10]. Studies first went through a title and abstract review. Those deemed eligible went through further full‐text review. As defined in the protocol, any disagreement at each stage was resolved through consensus.

We extracted standardized data from each study including study design, study cohort, geographic location (ISO1‐ISO3, where applicable), dates of data collection (i.e., the study period), incidence rate (including 95% confidence intervals (CI) and standard errors (SE)), cumulative incidence (including 95% CI and SE), person‐years at risk, number of HIV seroconversions, number of individuals in the study overall and by sex and age where applicable, total numbers of HIV‐negative and ‐positive participants at baseline and a description of the intervention if the study was a randomized controlled trial (RCT). Data from multicentre RCTs were disaggregated by site if possible. Incidence rates were further disaggregated by sex, age group/range, population risk group and intervention arm when available. Population risk groups included sex workers (SW), MSM, transgender women (TGW), pregnant women (PW), serodiscordant couples (SDC), Lake Victoria fisherfolk (FF), other high‐risk populations (e.g., women with multiple sexual partners; bar workers) and general populations (i.e., geographically defined populations with no defining characteristics beyond location of residency). For studies measuring cross‐sectional incidence through either BED capture enzyme immunoassay (BED‐CEIA) or limiting antigen avidity (LAg‐Avidity) enzyme immunoassay, we also recorded information on the window period, false recency rate and optical density (OD) reported.

For incidence rates, all estimates were converted to number of seroconversions per 100 pys at risk. We did not assess study quality or perform a risk‐of‐bias assessment for three reasons: (a) rates of HIV incidence were expected to vary across geography and between risk groups; (b) our primary objective was to summarize incidence data as opposed to assessing the causal impact of any particular intervention or exposure; and (c) there was substantial heterogeneity in the methods used to obtain incidence estimates.

### 2.2 Statistical analysis

Studies were grouped into geographic regions as defined by the UN Statistics Division [11]. However, Malawi, Mozambique, Zambia and Zimbabwe were categorized as part of the southern region due to the similarity of their epidemic, most notably subtype distribution, with other countries in those respective regions.

We summarized HIV incidence trends across general population studies over calendar time for eastern and southern Africa by using weighted LOESS regression for studies in which the calendar midpoint of the data collection period occurred on or after 8 October 2006 (i.e., the ≥25th percentile of the distribution of data collection calendar midpoints). Weights corresponded to the study size (*N*) of the population. In order to estimate the rate of change in incidence since 2010, we fit weighted linear regression models weighted by study size for all studies in which the calendar midpoint of the data collection period was in 2010 or later. For countries where study size was not reported for a specific time point, the most recent study size reported either at follow‐up or baseline was used. All trend analyses were stratified by sex. We excluded studies that only reported incidence estimates for men and women combined, age‐specific incidence estimates for age bins less than 26 years and studies that reported incidence estimates for nongeneral population subgroups. The 26‐year cutoff was chosen because it was the smallest age bin including more than two thirds (>70%) of the adult population most at risk for HIV, typically defined as 15 to 49 years old. For studies where CIs were not reported, we derived exact CI based on a Poisson distribution. Studies that did not report an upper or lower age limit were included (e.g., studies that reported a minimum enrolment of 15 years but no max age enrolment), but these studies were excluded in sensitivity analyses. Point estimates were plotted at the midpoint of the data collection period (e.g., if a study was conducted from 1 January 2010 to 31 December 2010, the study midpoint would be 2 July 2010), with bars indicating the entire length of the collection period (e.g., 1 January 2010 through 31 December 2010). Trends in HIV incidence were not examined for western or central Africa or for other population risk groups given limited availability of incidence data.

We further summarized recent incidence trends over the last decade within study cohorts over time using linear regression models. This analysis was limited to studies that reported incidence estimates for at least three unique calendar periods since 1 January 2010. For studies that reported age‐stratified estimates, we used incidence metrics for the widest age bin reported, but did not restrict based on age‐bin size.

We constructed forest plots to summarize HIV incidence rates and 95% CI by region and risk group. For studies where incidence estimates were reported for multiple time periods, we only included the most recent estimate. Similarly, if multiple studies reported estimates from the same cohort, we used the most recent estimate available. For RCTs, we included the estimate from the control arm only with the exception of studies of hormonal contraceptive use, in which case the overall estimate was used. For studies reporting both national and sub‐national incidence data, estimates at the national level only were included. For general population studies, we only reported incidence estimates for age bins spanning 26 years or greater.

All analyses were conducted in R (version 4.0.3) and Stata (version 14).

## 3 RESULTS AND DISCUSSION

**Figure 1:**
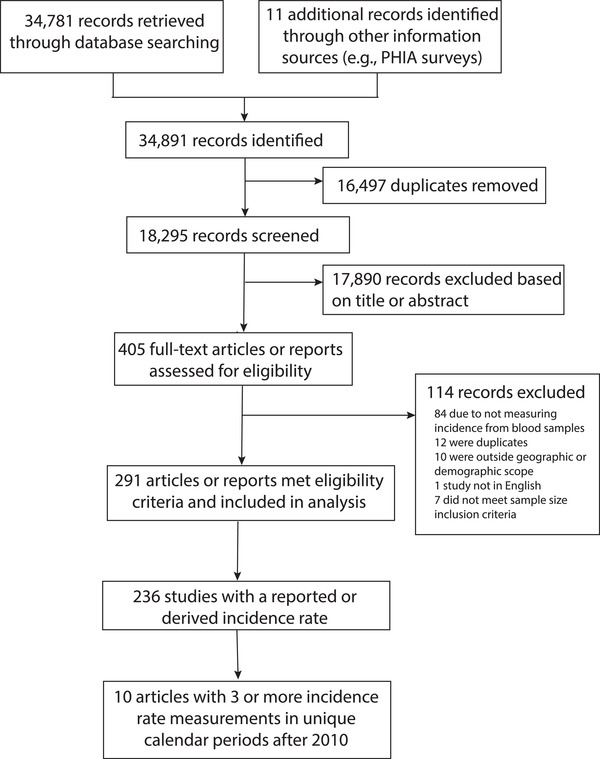
Flow of papers from literature search to full‐text review. Total 34,781 papers published between 2010 and 2019 were identified through PubMed, Embase, Scopus and OVID global health, and 11 additional papers were identified through alternative sources including population‐based HIV impact assessment (PHIA) surveys. Of these, 16,497 duplicates were removed using Covidence. Of the 18,295 studies screened, 291 papers met eligibility criteria and were included in the analysis.

There were 34,781 records retrieved through the database searches and an additional 11 technical reports identified and included. After duplicates were removed, 18,295 records remained. Of these, 17,890 records were excluded after title and abstract screening, with 405 records remaining for full‐text review. Of these, 114 studies were excluded due to not meeting prespecified inclusion criteria. There were 291 studies that reported some form of HIV incidence data (e.g., cumulative incidence, number of seroconversions, incidence rate), and 236 for which at least one incidence rate estimate was reported or could be derived (*n = 225 reported and n = 11 derived*; Figure 1; Table S1).

### 3.1 Characteristics of included studies

**Figure 2:**
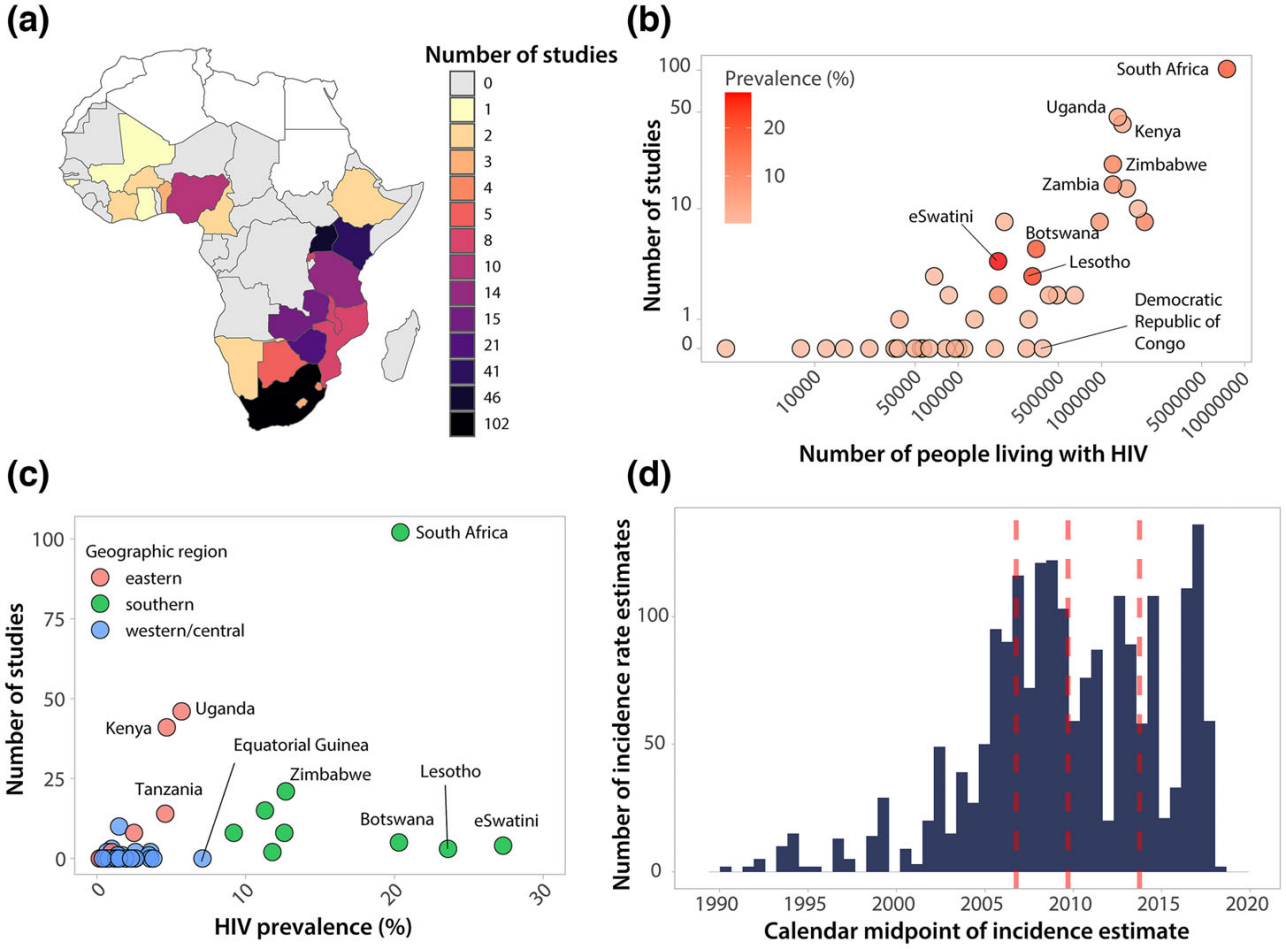
Number of studies reporting empirical HIV incidence data published between 2010 and 2019 by location, HIV burden and study period. (a) Number of studies by country in sub‐Saharan Africa. Studies only reporting incidence data across countries (e.g., South Africa and Zambia combined) were not included. (b) Number of studies by number of people living with HIV. Number of people living with HIV taken as the mean estimated number of adults (15+) living with HIV in 2018 from UNAIDS. (c) Number of studies by HIV prevalence and region. Prevalence taken as the 2018 adult (15 to 49) prevalence estimate from UNAIDS. (d) Distribution of calendar midpoints for HIV incidence rate estimates. The median and interquartile ranges of calendar midpoints are shown in red.

The last comprehensive systematic review of the empiric HIV incidence literature in sub‐Saharan Africa was published in 2009 and included 57 studies conducted in 14 sub‐Saharan African countries [9]. In this systematic review of incidence data published between 2010 and 2019, 291 studies reported incidence data from 22 sub‐Saharan countries (Figure 2a). Most studies were conducted in South Africa (*n* = 102), Uganda (*n* = 46), Kenya (*n* = 41), Zimbabwe (*n* = 21) and Tanzania (*n* = 14). Of note, there were 43 incidence estimates reported from multicountry studies (e.g., studies reporting combined estimates from two or more countries). While the number of studies published per country correlated with total case burden (Figure 2b), there were 26 countries in sub‐Saharan Africa with no published incidence data. The majority of these countries were in western and central Africa (62%; *n* = 16), illuminating critical geographic gaps in surveillance and interventional research efforts. Overall, only 20 (7%) studies were conducted in western and central Africa, despite this region accounting for an estimated 20% of all incident cases in sub‐Saharan Africa in 2019.^1^

**Figure 3:**
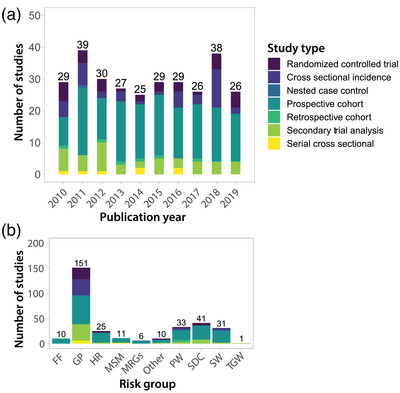
Number of studies reporting empirical HIV incidence data by year and risk group. (a) Number of studies published by calendar year broken down by study type. (b) Number of studies published by risk group broken down by study type. FF, fisherfolk; GP, general population; HR, high risk (e.g., women who report multiple partners); MSM, men who have sex with men; MSRG, multiple risk groups (e.g., SW and MSM); PW, pregnant women; SDC, serodiscordant couples; SW, sex workers; TGW, transgender women.

Figure 2c shows the number of studies reporting incidence data versus the prevalence of infection at the country level using 2018 national prevalence estimates from UNAIDS [12]. Of the five countries with the highest HIV prevalence, there were only two with more than 10 studies published since 2010 (South Africa with 102 estimates and an HIV prevalence of 20.4%, Zimbabwe with 21 estimates and a prevalence of 12.7%). The remaining three countries, eSwatini, Lesotho, and Botswana, had prevalences of 27.3%, 23.6% and 20.3%, but only had four, three and five incidence studies published, respectively (Figure 3a). While the number of studies conducted per country tended to trend with higher HIV case burden and prevalence, and a lack of empiric incidence data in western and central Africa may be in part because of lower regional incidence, there were several countries with either moderately high case burden or prevalence reporting no or very limited data, including the Democratic Republic of Congo and Equatorial Guinea. Similar geographic disparities in incidence data have been reported earlier [9].

Publication of incidence data fluctuated little over calendar time (Figure 3a). Most incidence estimates were derived from prospective observational cohort studies (*n* = 163), followed by secondary analyses of RCTs (*n* = 46), cross‐sectional incidence studies (*n* = 41), primary analyses of RCTs (*n* = 34), serial cross‐sectional studies (*n* = 7), retrospective cohort studies (*n* = 6) and nested case control studies (*n* = 1). Of note, there were seven studies reporting incidence data from two design classifications. Figure 3b shows studies disaggregated by population risk group. One hundred fifty‐one estimates were drawn from GP studies, 41 from SDC, 33 from PW, 31 from SW, 11 from MSM and 10 from FF communities in eastern Africa.

### 3.2 HIV incidence trends over calendar time in eastern and southern Africa

**Figure 4:**
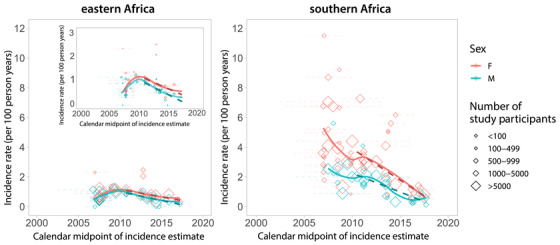
HIV incidence rates in general population studies over time by sex for eastern and southern Africa. We included incidence rate estimates from general population studies in southern and eastern Africa that had a study midpoint from 2007 onwards (this corresponds to the 25th percentile of all calendar midpoints reported, Figure 2d). Diamonds represent the calendar midpoint of the incidence rate estimate, while error bars represent the start and end date of the time interval over which the incidence rate was measured. Estimates are only shown for studies with an age range spanning 26 years or greater (e.g., an HIV estimate for individuals 18 to 44 years). Dashed lines show incidence trends fit using linear regression. Solid lines represent smoothed curves fit using LOESS regression. An inset is included for eastern Africa to highlight trends with a *y*‐axis restricted to three per 100 person‐years.

Next, we summarized HIV incidence trends over calendar time for GP studies by sex and geographic region (Figure 4). A total of 48 studies contributed 142 estimates (*n* = 80 for women; *n* = 62 for men) to the analysis. We observed steadily declining HIV incidence rates across general population studies in eastern and southern Africa since 2010. While HIV incidence was consistently higher among women than men, there were reductions in incidence rates observed in both sexes. In eastern Africa, annual incidence declined by 0.12/100 pys (95% CI: 0.06–0.18; *p* = 0.001) among men and by 0.10/100 pys (95% CI: −0.02–0.22; *p* = 0.093) among women. HIV incidence fell more rapidly in southern Africa, declining by 0.25/100 pys (95% CI: 0.17–0.34; *p* < 0.0001) annually among men and by 0.42/100 pys (95% CI: 0.23–0.62; *p* = 0.0002) annually among women. In sensitivity analyses, we excluded studies that did not report either an upper or lower age range (*n* = 15) and observed similar trends (Figure S1). We also compared incidence trends estimated from directly observed data in eastern and southern Africa combined to UNAIDS modelled trends for the same region. While both empirical and modelled trends showed declining incidence in eastern and southern Africa since 2010, empiric incidence rates were relatively higher overall and declined more precipitously (Figure S2). These broad reductions in HIV incidence are likely the partial result of massive global efforts to rollout and scale‐up HIV prevention and treatment interventions, including antiretroviral therapy (ART) and voluntary medical male circumcision (VMMC). Indeed, several population‐based cohorts and RCTs have reported on the link between ART and VMMC and declines in population‐level incidence [13, 14, 15, 16, 17, 18, 19].

### 3.3 HIV incidence trends over calendar time within study cohorts

**Figure 5:**
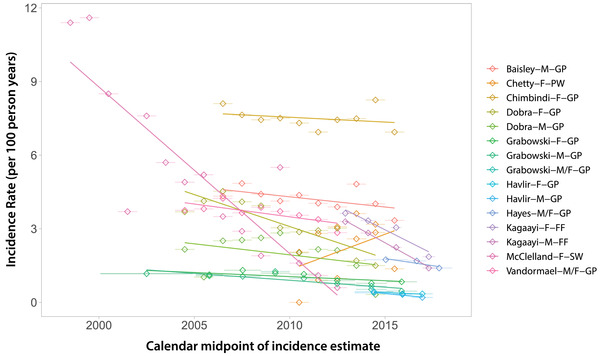
HIV incidence trends from 10 studies with three or more incidence rate measurements after 2010. Data are shown overall and disaggregated by sex where possible. The legend shows the first author, sex (M, male; F, female) and risk group (GP, general population; PW, pregnant women; FF, fisherfolk; SW, sex worker).

We also examined HIV incidence trends within studies that had more than three incidence rate measurements in unique calendar periods since 1 January 2010 (Figure 5). Of the 291 studies, only 38 (13%) studies reported incidence rate estimates for more than one unique calendar period (Figure S3), and of these studies only 10 had three or more measurements since 2010. Within nine of these studies, a decline in HIV incidence was observed over calendar time consistent with our analyses of incidence across general population studies. Of note, two of these nine studies were conducted in high‐risk key populations, one among Lake Victoria fisherfolk and the other among SW [13, 20], suggesting incidence reductions may not be exclusive to general population cohorts. The single outlier study showed rising incidence among pregnant women in South Africa [21].

### 3.4 Geographical variation in HIV incidence estimates

**Figure 6:**
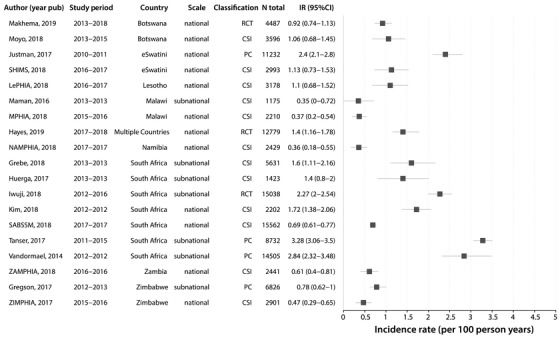
Forest plot of HIV incidence estimates after 2010 for general population studies in southern Africa. Only the most recent HIV incidence estimate for a cohort/study population is shown. Incidence rates are reported as the number of new cases per 100 person‐years and the error bars represent 95% CI. Study references are reported in Table S1. RCT, randomized controlled trial; CSI, cross‐sectional incidence study; PC, prospective cohort.

Within eastern and southern Africa, we found evidence of substantial geographic variation in HIV incidences rates, consistent with an earlier mapping study reporting marked small‐scale spatial heterogeneities in disease burden across the continent [22]. Figure 6 summarizes the most recent HIV incidence estimates since 2010 for GP studies in southern Africa. National estimates ranged from 0.37 per 100 pys in Malawi (2015 to 2016) to 2.4 per 100 pys in eSwatini (2010 to 2011). However, by 2016 to 2017, HIV incidence declined to 1.13 per 100 pys in eSwatini.

There was substantial variation across national and sub‐national incidence estimates in South Africa, with rates ranging from 0.69 at the national level to 3.28 per 100 pys sub‐nationally. Of the four measurements exceeding two per 100 pys, three were in South Africa and one in eSwatini. Overall, South Africa reported the highest rates of HIV incidence in the general population, and these estimates were predominately concentrated in KwaZulu‐Natal, a coastal province in the southeast. Prior studies in South Africa have identified high‐risk corridors of transmission with elevated HIV incidence rates compared to the surrounding population [23]. Furthermore, these areas tended to correlate with higher prevalence, suggesting that current maps of HIV prevalence, potentially in combination with uptake of HIV prevention and treatment data, could be used to strategically implement HIV programmes and target interventions, such as pre‐exposure prophylaxis (i.e., PrEP), to high‐risk populations. Community viral load data have also been shown to be important indicators of local HIV incidence in large RCTs of universal HIV treatment [24].

**Figure 7:**
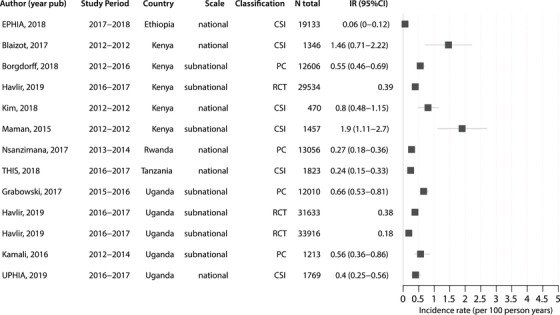
Forest plot of HIV incidence estimates after 2010 for general population studies in eastern Africa. Only the most recent HIV incidence estimate for a cohort/study population is shown. Incidence rates are reported as the number of new cases per 100 person‐years and the error bars represent 95% CI. Estimates without error bars did not report a confidence interval/standard error for the estimate. Study references are reported in Table S1. PC, prospective cohort; CSI, cross‐sectional incidence study; RCT, randomized controlled trial.

Figure 7 shows the most recent HIV incidence estimates since 2010 for GP studies in eastern Africa. Estimates ranged from 0.06 per 100 pys (95% CI: 0–0.12) in Ethiopia to 1.9 per 100 pys (95% CI: 1.11–2.7) in Kenya. There was substantial sub‐national variation in HIV incidence estimates within Kenya, with rates ranging between 0.55 and 1.9 per 100 pys, although lower incidence rates tended to be observed in more recent years. National studies were conducted in Ethiopia, Kenya, Rwanda, Uganda and Tanzania, and all HIV incidence estimates were below 1 per 100 pys. There were only two general population studies in west Africa since 2010, including the Cameroon PHIA in 2017 to 2018, which reported an HIV incidence rate of 0.27 (95% CI: 0.14–0.41) per 100 pys [25], and another study in blood donors in Cote d'Ivoire (0.07 per 100 pys; 95% CI: 0.06–0.08) [26].

### 3.5 HIV incidence among key populations

We collated recent HIV incidence estimates for key populations, including SW, MSM and FF (Figures S4‐S6). Overall, there were few incidence estimates available for these groups since 2010; however, incidence rates in these populations were typically much higher than those observed in GP studies in line with prior systematic reviews of HIV prevalence data [27, 28]. All estimates for SW (*n* = 7) were derived from cohort studies and ranged from 0.6 to 3.4 per 100 pys (Figure S4). Incidence estimates among MSM cohorts (*n* = 5) were highly variable ranging from 1.03 per 100 pys (95% CI: 0.03–5.79) in Kenya to 15.4 per 100 pys (95% CI: 12.3–19) in Nigeria (Figure S5). Among FF populations, HIV incidence ranged from 1.59 to 6.04 per 100 pys (Figure S6).

### 3.6 Sex differences in HIV incidence estimates within general population studies

**Figure 8:**
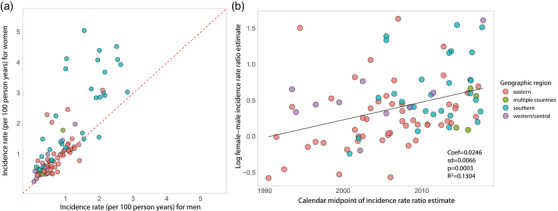
HIV incidence rates among men and women in general population studies. (a) Plot of male versus female HIV incidence per 100 person‐years. Dashed red line is the identity line; points above the line represent higher female HIV incidence, while points below the line represent higher male HIV incidence. (b) Scatterplot of the log female:male HIV incidence ratio over calendar time with fitted line (solid black line) estimated using linear regression.

We also directly compared HIV incidence estimates between men and women within GP studies (Figure 8). Of the 236 studies with incidence rates reported, 108 (46%) reported incidence estimates for women only and 18 (6%) for men only. There were an additional 23 (10%) studies that reported combined estimates for men and women only, and 87 (37%) studies reported sex‐stratified results. HIV incidence was typically higher among women in line with a recent systematic review of incidence among African adolescents and young adults 15 to 24 years old [29]. Among 43 GP studies reporting 95 sex‐stratified incidence estimates for age bins ≥26 years, female HIV incidence was higher in 77 (81%) instances and the median female‐to‐male HIV incidence ratio was 1.47 (IQR: 1.11 to 1.83) overall (Figure 8a). A better understanding of incidence and associated risk factors for transmission among men might potentially result in lower female transmission given the large preponderance of heterosexual HIV transmission in Africa [1]. Studies throughout Africa show lower uptake of HIV prevention and treatment interventions among men, and also lower participation rates in HIV incidence studies [30].

Lastly, temporal assessment of the female‐to‐male incidence rate ratio showed evidence for growing disparity by sex over calendar time, increasing by 1.02% per year on average (95% CI: 1.01–1.04%; *p* = 0.0003; Figure 8b). While Figure 4 appears to show a lack of divergence in incidence between sexes, parallel trends on the linear scale are reflective of a shifting ratio overtime. Indeed, a plot of these same data log‐transformed shows evidence of increase in the female‐to‐male incidence ratio, particularly in eastern Africa (Figure S7). It is unclear what is driving this growing HIV disparity between men and women; however, lower male susceptibility due to increasing VMMC coverage and higher female ART uptake may partially explain this observation.

### 3.7 Study limitations

There are limitations to this study. First, the methods used to obtain HIV incidence estimates across studies including study design, testing algorithms and definitions of incidence were highly variable. Second, upper and lower age eligibility criteria were often unreported. In the case of general population studies that did not report an upper or lower age bound, we imputed the range to be 15 to 65 years. In a sensitivity analysis assessing incidence trends over calendar time, we excluded these studies and found no qualitative differences in our inferences. Third, there were many countries and geographic locations for which there were no incidence data available. It is unclear whether the data presented here are representative of empirical trends in unobserved locations, most notably western and central Africa. Reassuringly, our results are consistent with UNAIDS regional models of incidence trends also showing declines in new cases from 2010 to 2020 [31]. While UNAIDS models partly rely on empiric incidence data to reconstruct incidence trends, including cross‐sectional incidence estimates from PHIA surveys, they are predominately informed by prevalence rather than incidence data, and directly observed incidence trends from cohort studies are not included (personal communication: Dr. Jeff Eaton). Of note, the HIV incidence rates and declines in incidence over calendar time observed in this analysis of empirical data were greater than those compared to UNAIDS modelled incidence trends from the same region. However, this is expected, as HIV research efforts are typically concentrated in higher incidence areas and likely subjected to more intensive intervention. Fourth, our analyses of incidence trends were only done among general population cohorts and thus may not reflect changes among key populations. Fifth, published data may not be fully representative of all incidence data, particularly studies with higher incidence rates may be more likely to be published than those with low or zero incidence. Sixth, this systematic review included nine cross‐sectional studies that relied on the BED capture immunoassay (BED‐CEIA) to measure incidence, including two general population studies that met inclusion criteria for Figure 4. While the BED‐CEIA has been shown to systematically overestimate HIV incidence in validation studies [32], exclusion of these studies in sensitivity analyses did not impact observed incidence trends (data not shown). Next, this study only includes data published through 2019, and incidence trends observed here may not reflect more recent disease dynamics. While UNAIDS estimates that HIV incidence has continued to decline through 2020 (−31% since 2010), trends may shift in the coming years due to the ongoing COVID‐19 pandemic. Modelling studies have found that even short‐term interruptions in HIV treatment and prevention services, including suspension in HIV testing programmes, could lead to substantial increases in HIV‐related deaths and new HIV infections [33, 34]. Indeed, a 2021 Global Fund survey found significant recent disruptions to HIV prevention services due to COVID‐19 [35]. Lastly, the results of this study may have been impacted by our own review methodologies, including the search strategy and our inclusion and exclusion criteria. Critically, we excluded studies that were not in English, which may explain some of the data gaps observed in western and central Africa.

### 3.8 Summary of findings and public health implications

Monitoring HIV incidence trends is essential for assessing progress and targeting resources. Our analysis of directly observed HIV incidence data demonstrates that the rate of new infections is declining in eastern and southern Africa among both men and women. These findings are consistent with UNAIDS incidence models, which have also shown reductions in new cases continent‐wide over the last decade [1, 6], although the overall levels of incidence and rates of decline in incidence were greater in this analysis of empiric data. Critically, major gaps exist in the availability of empiric HIV incidence data, particularly for western and central Africa and among key populations, including MSM, among whom HIV incidence was highest. HIV incidence remains higher among women, despite overall reductions in acquisition risk in both sexes, and the incidence gap between sexes appears to be growing with time.

Taken together, our findings provide empirical evidence that HIV incidence in eastern and southern Africa has declined over the last decade. The widespread scale‐up and uptake of HIV treatment and prevention interventions have been linked to incidence declines within individual studies previously [13, 14, 15, 16, 17, 18, 19], and are likely contributing to the broad‐scale incidence reductions observed here. Sustained commitment to these programmes and new investment in innovative technologies and approaches may ultimately help achieve HIV elimination goals in sub‐Saharan Africa. However, future successes remain in the balance as global funding for HIV programmes in low‐ and middle‐income countries dwindles [2]. The COVID‐19 pandemic presents further challenges as critical resources are diverted and mobility restrictions impact uptake of interventions, in particular, HIV testing and timely initiation of ART [34, 36]. Ongoing surveillance, including addressing geographic and population disparities in HIV incidence data, will be critical to maintaining and building upon past success in the coming decade.

## 4 CONCLUSIONS

Our review of directly observed HIV incidence estimates shows evidence that HIV incidence is declining among general populations of eastern and southern Africa, consistent with UNAIDS mathematical models of HIV incidence trends through 2020 [31]. However, empiric data from western and central Africa and among key populations, including MSM, were relatively rare; thus, declining trends in general populations in eastern and southern Africa may not reflect trends in other populations. Such critical gaps in incidence data have persisted since the onset of the HIV pandemic, despite significant disease burden among western and central African countries and key populations, requiring a call to action to improve surveillance efforts and representativeness in clinical trials. HIV incidence remains higher in women than men, for reasons not fully explained, and this disparity will need to be addressed to achieve HIV control and elimination targets set forth by the global public health community. Targeted outreach to men most likely to transmit and women at highest risk of HIV, perhaps through long‐acting injectable treatment and prevention, may be an effective strategy, but additional research will be needed to identify these sub‐groups across a rapidly changing epidemic landscape. Ongoing HIV incidence surveillance, through both empiric and model‐based approaches, is critical for measuring continued impact of HIV control programmes and potential disruptions due to COVID‐19.

## COMPETING INTERESTS

The authors declare that they have no competing interests.

## AUTHORS’ CONTRIBUTIONS

M.K.G., K.J., J.L. and A.T. designed this study. K.J., O.O., S.B. and G.L. screened, reviewed and extracted data. K.J. conducted the statistical analysis. K.J. and M.K.G. wrote the first draft of the manuscript. All authors reviewed and approved the final draft.

## Supporting information


**Table S1**. Full list of the 292 studies reporting HIV incidence data

**Figure S1**. Sensivity analysis of time trends excluding studies that did not report a minimum or maximum age range for the study
**Figure S2**. Comparison of HIV incidence trends by sex in eastern and southern Africa derived from directly observed HIV incidence data or UNAIDS models
**Figure S3**. 38 studies reporting two or more incidence rate estimates in unique calendar periods
**Figure S4**. Forest plot of HIV incidence estimates after 2010 for studies among female sex workers (SW)
**Figure S5**. Forest plot of HIV incidence estimates after 2010 for studies among men who have sex with men (MSM)
**Figure S6**. Forest plot of HIV incidence estimates after 2010 for studies of Lake Victoria fisherfolk in Eastern Africa
**Figure S7**. Log‐transformed HIV Incidence rates in general population studies over time by sex for eastern and southern Africa
