# Supplementary material for: Declining HIV incidence in sub‐Saharan Africa: a systematic review and meta‐analysis of empiric data
Source: J Int AIDS Soc. 2021 Oct 20;24(10):e25818. doi: 10.1002/jia2.25818 (PMC8528667; doi:10.1002/jia2.25818)
Supplement: Supplementary file 2 — Figure S1. Sensivity analysis of time trends excluding studies that did not report a minimum or maximum age range for the study Figure S2. Comparison of HIV incidence trends by sex in eastern and southern Africa derived from directly observed HIV incidence data or UNAIDS models Figure S3. 38 studies reporting two or more incidence rate estimates in unique calendar periods Figure S4. Forest plot of HIV incidence estimates after 2010 for studies among female sex workers (SW) Figure S5. Forest plot of HIV incidence estimates after 2010 for studies among men who have sex with men (MSM) Figure S6. Forest plot of HIV incidence estimates after 2010 for studies of Lake Victoria fisherfolk in Eastern Africa Figure S7. Log‐transformed HIV Incidence rates in general population studies over time by sex for eastern and southern Africa [file JIA2-24-e25818-s002.docx]

**Supplemental Figures**


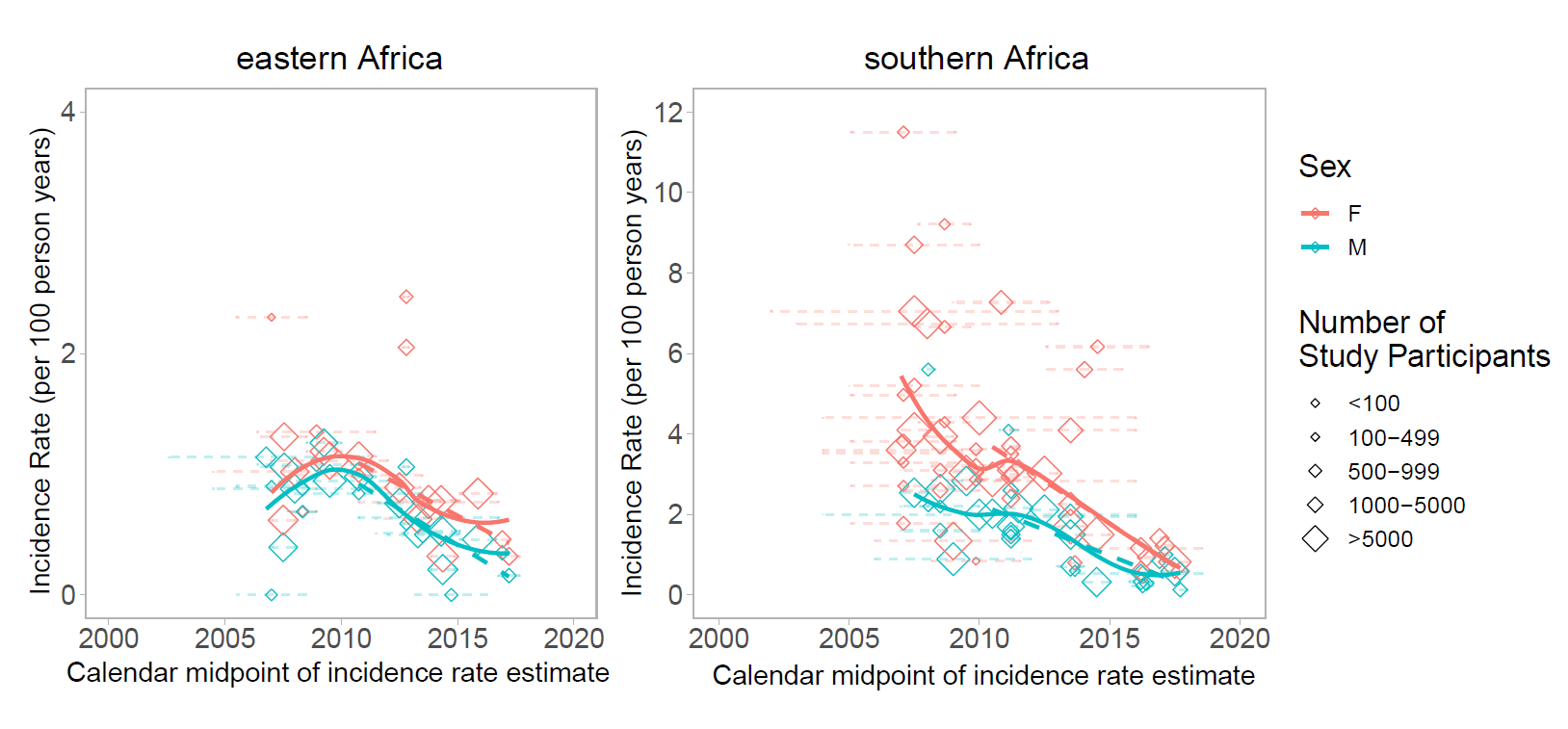


**Supplemental Figure 1**. Sensivity analysis of time trends excluding studies that did not report a minimum or maximum age range for the study.

**
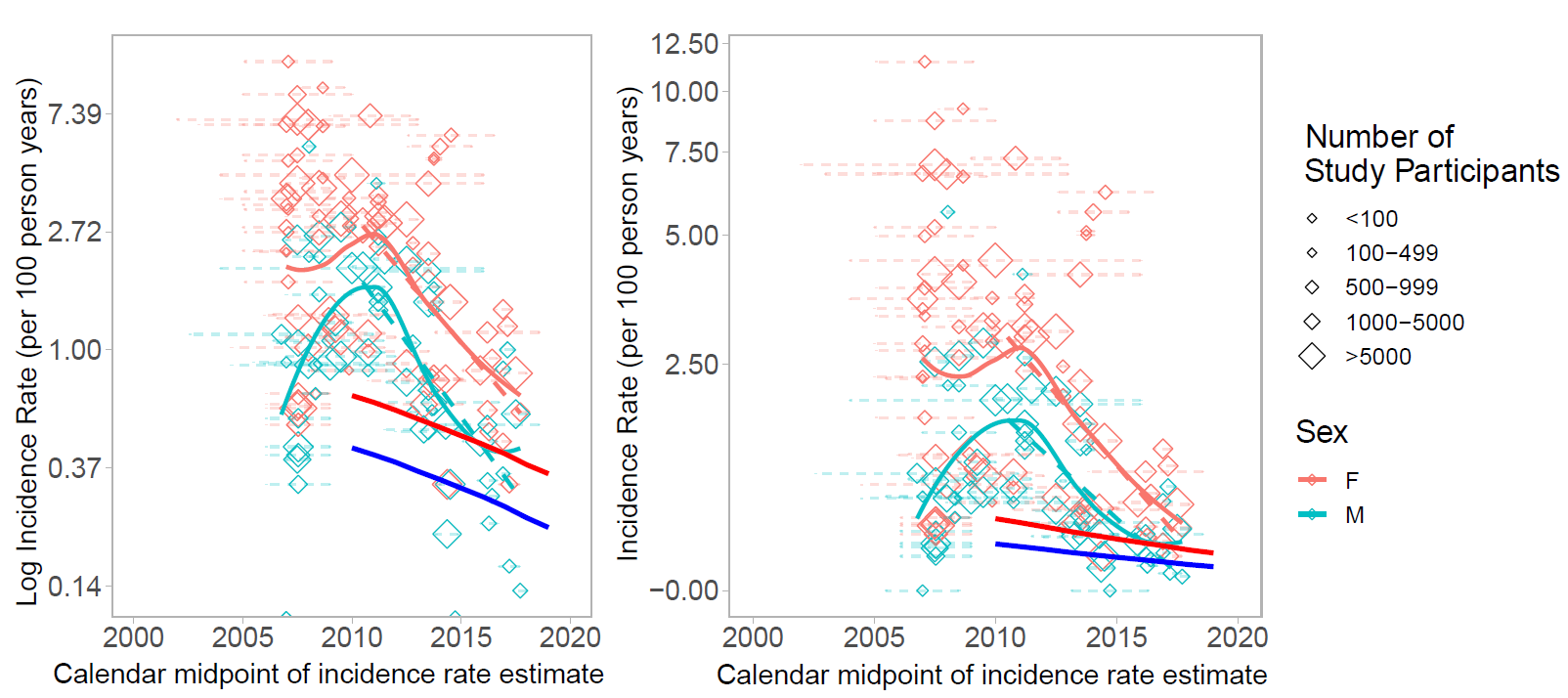
**

**Supplemental Figure 2.**Comparison of HIV incidence trends by sex in eastern and southern Africa derived from directly observed HIV incidence data or UNAIDS models. UNAIDS data were obtained from [https://aidsinfo.unaids.org/](https://nam02.safelinks.protection.outlook.com/?url=https%3A%2F%2Faidsinfo.unaids.org%2F&data=04%7C01%7Cmgrabow2%40jhu.edu%7C45d123a4c472409c179a08d91a1b9a35%7C9fa4f438b1e6473b803f86f8aedf0dec%7C0%7C0%7C637569528118095319%7CUnknown%7CTWFpbGZsb3d8eyJWIjoiMC4wLjAwMDAiLCJQIjoiV2luMzIiLCJBTiI6Ik1haWwiLCJXVCI6Mn0%3D%7C1000&sdata=1p7CNZQgPqM9%2FU%2BLGyVY%2Fx7WQKgBY8iqouVeXm5i%2FkU%3D&reserved=0). Panel**(A)**shows HIV incidence rates plotted on the log scale, while panel **(B)**shows HIV incidence rates transformed to the pseudo-log scale. Smoothed curves were fit to empiric data and were fit using loess regression in R. Note that studies with HIV incidence rates of 0 (n=2, both from eastern Africa) are excluded from this figure.


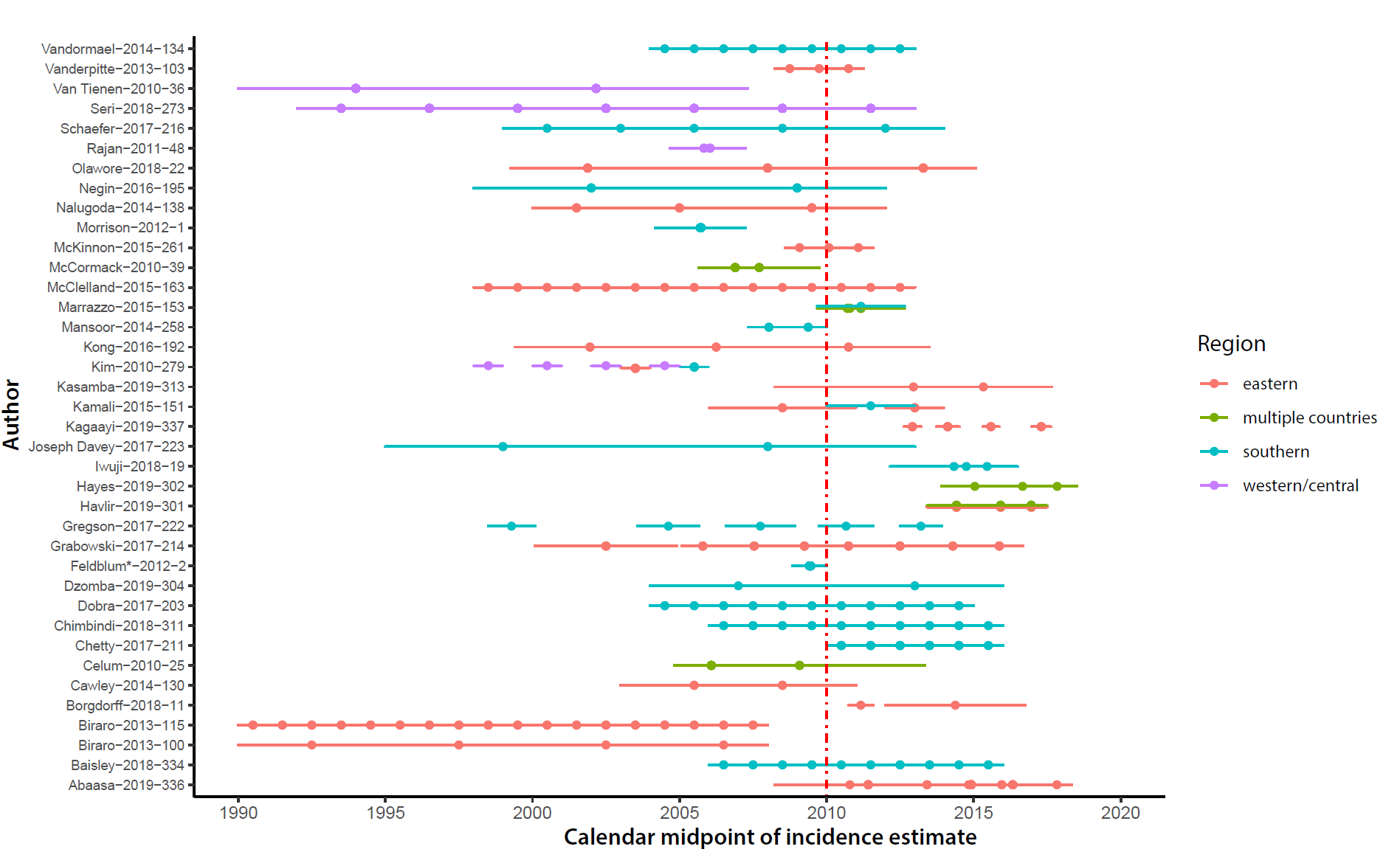


**Supplemental Figure 3. 38 studies reporting two or more incidence rate estimates in unique calendar periods**. The dots represent the calendar midpoint of the incidence estimate and the lines the time period over which incidence was measured. *This study’s prior incidence estimate was in 1993 and is not shown.


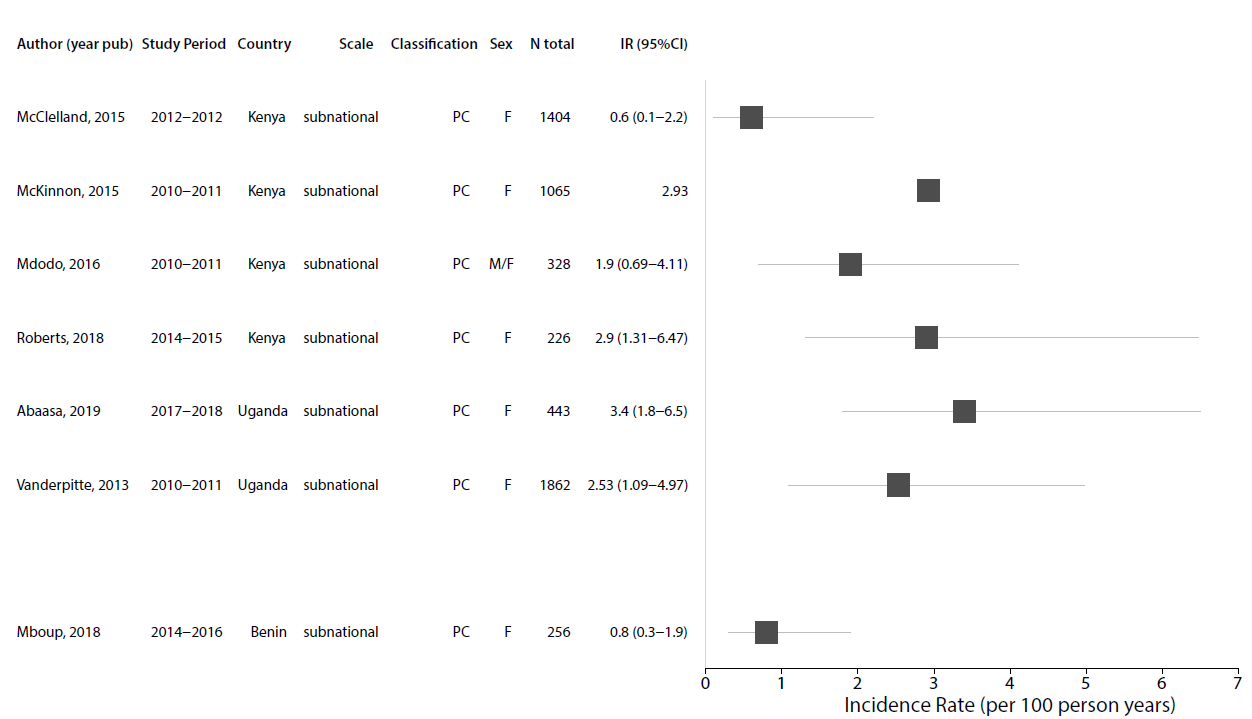


**Supplemental Figure 4. Forest plot of HIV incidence estimates after 2010 for studies among female sex workers (SW)**. Only the most recent HIV incidence estimate for a cohort/study population are shown. Incidence rates are reported as the number of new cases per 100 person-years and the error bars represent 95% CI. Estimates without error bars did not report a confidence interval/standard error for the estimate. Study references are reported in supplemental Table 1. PC=prospective cohort; SCS=serial cross-sectional study


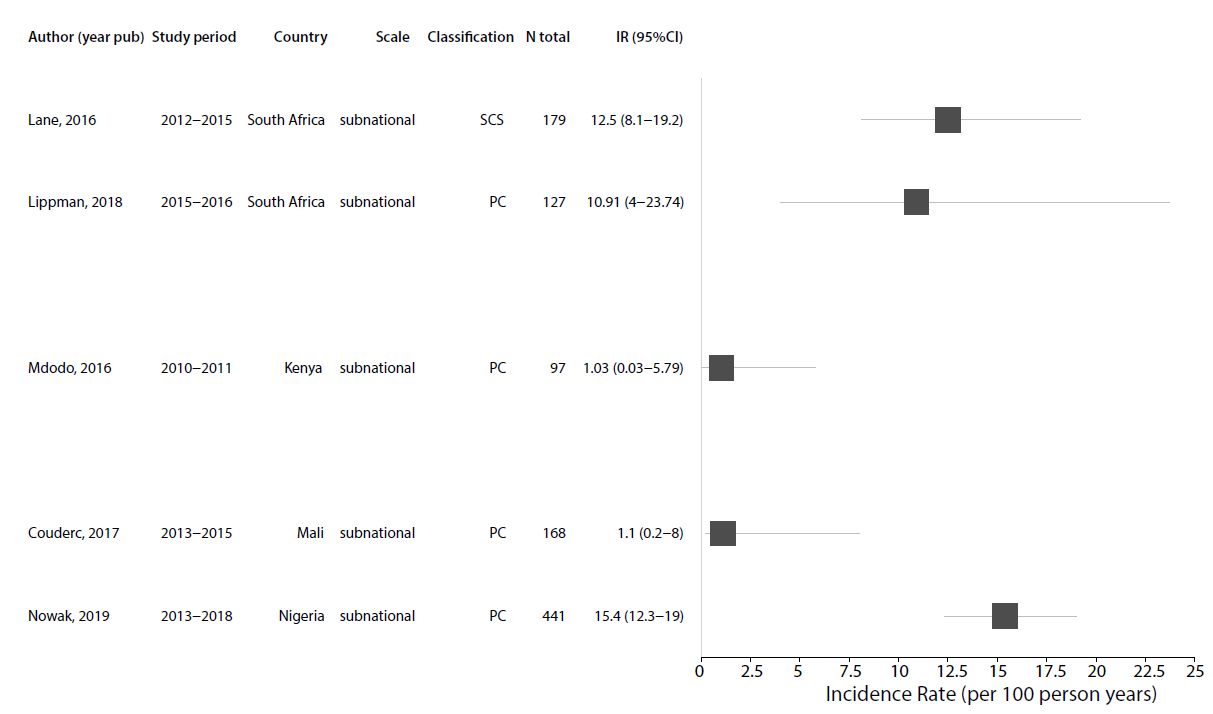


**Supplemental Figure 5. Forest plot of HIV incidence estimates after 2010 for studies among men who have sex with men (MSM)**. Only the most recent HIV incidence estimate for a cohort/study population are shown. Incidence rates are reported as the number of new cases per 100 person-years and the error bars represent 95% CI. Estimates without error bars did not report a confidence interval/standard error for the estimate. Study references are reported in supplemental Table 1. PC=prospective cohort; SCS=serial cross-sectional study


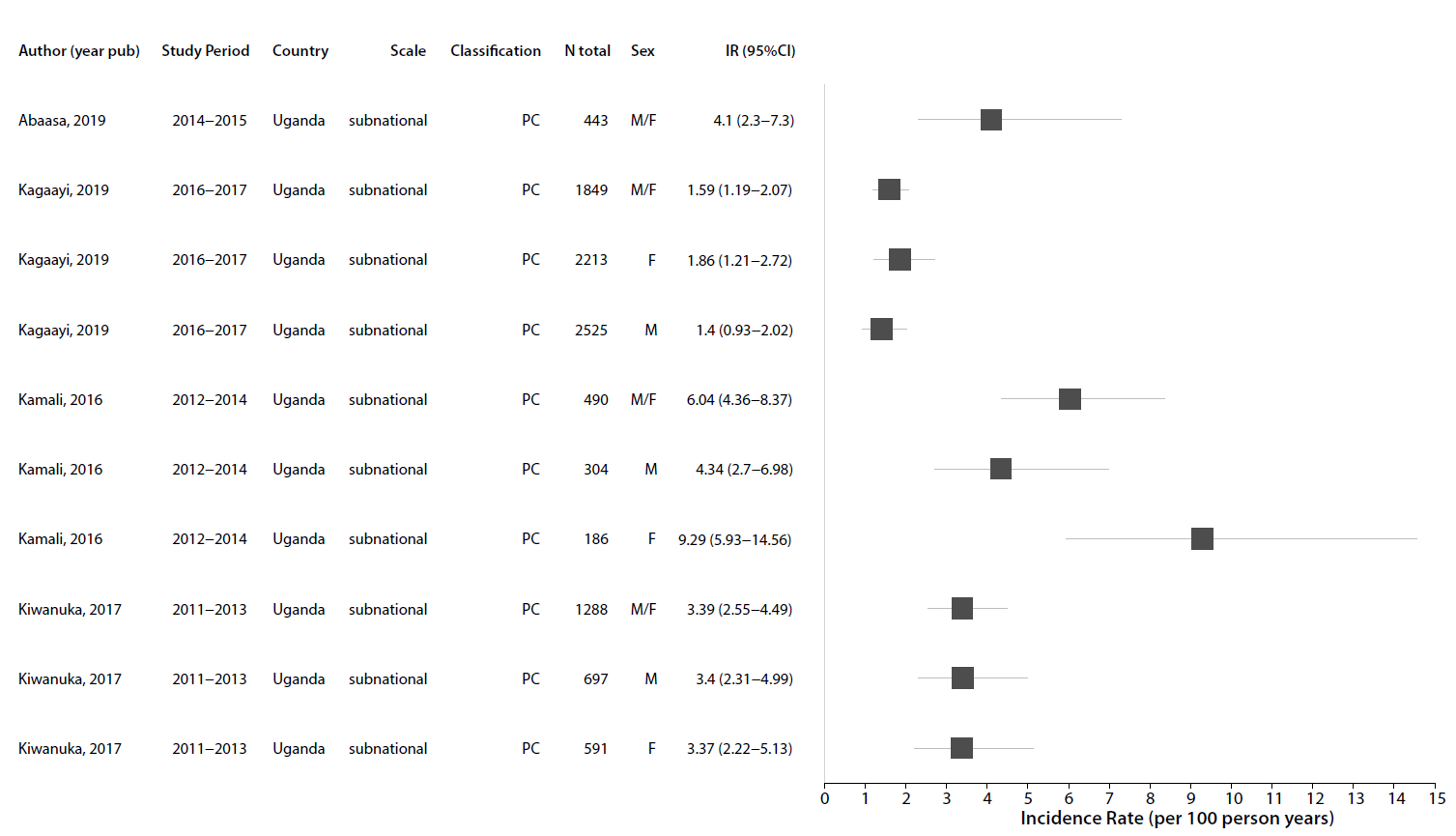


**Supplemental Figure 6. Forest plot of HIV incidence estimates after 2010 for studies of Lake Victoria fisherfolk in Eastern Africa**. Only the most recent HIV incidence estimate for a cohort/study population are shown. Incidence rates are reported as the number of new cases per 100 person-years and the error bars represent 95% CI. Estimates without error bars did not report a confidence interval/standard error for the estimate. Study references are reported in supplemental Table 1. PC=prospective cohort


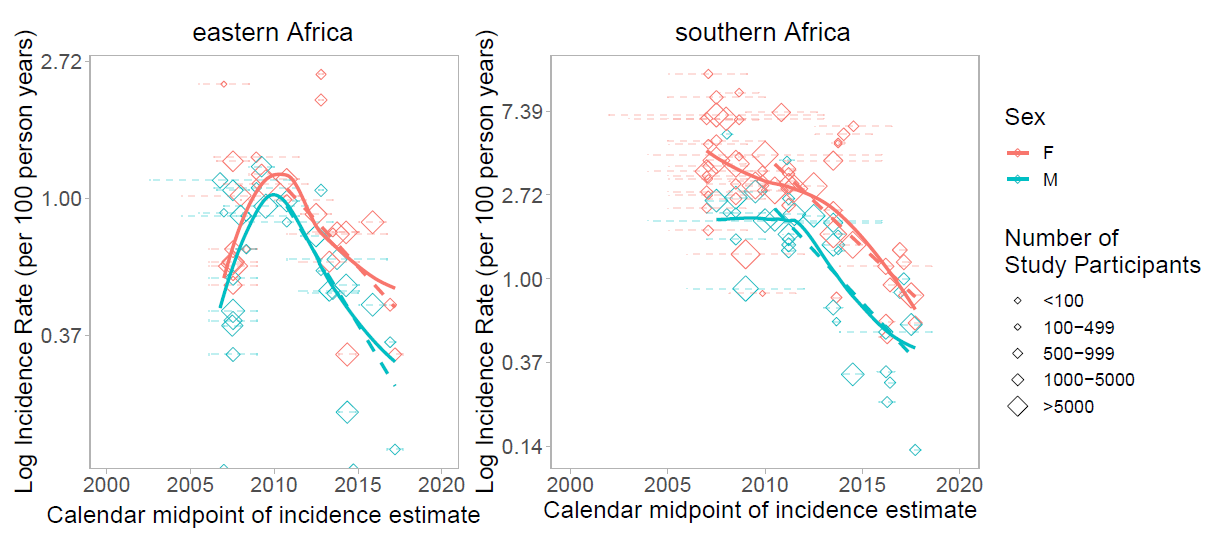


**Supplemental Figure 7: Log-transformed HIV Incidence rates in general population studies over time by sex for eastern and southern Africa**. Dashed lines show incidence trends fit using linear regression. Solid lines represented smoothed curves fit using loess regression. Note difference in Y axes limits for eastern and southern African plots.
